# Legal Notes for Institutional Workers

**Published:** 1915-07-24

**Authors:** 


### LEGAL NOTES FOR INSTITUTIONAL WORKERS.

### A Suggested Public Health Improvement.

In a recent number of the *American Law Review* there is an article containing a suggestion which we pass on to those interested in matters of public health. According to the law here at present, the discharge of refuse or sewage or chemicals into streams and rivers is practically left for regulation to the common law of nuisance—that is to say, unless the water is rendered offensive in a very distinctive degree to riparian owners or occupiers, no one has the right to interfere in the matter. From the article referred to it would appear that in certain of the United States the Public Health Board has an oversight of watercourses and a title to prohibit the use of them as receptacles for sewage or other discharge if in the opinion of the Board there is danger of injury to public health. Such a power seems to be a very reasonable function of a Public Health Authority, and one which, if properly exercised here, especially in the smaller towns and villages, should be a very valuable preventive of disease, provided it was nationally controlled, and not under the thumb of local interests and influences.

### The Fees of Medical "Expert" Witnesses.

The fees usually paid to skilled medical witnesses have for ordinary cases been settled by the practice of the Scottish Courts at £2 2s. for attendance at Court, with another £2 2s. for preliminary examination and report. In special circumstances the latter fee may be increased. In a recent case one of the medical experts claimed a larger fee on the ground that he was an *x*-ray specialist, and that he prepared two *x*-ray plates, which were produced at the trial along with the photographs printed from them. The Auditor of Court taxed his fee at £6 6s., thus increasing it beyond the normal figure, but on appeal the Sheriff reverted to the ordinary fee. We give part of his Lordship's opinion, which contains observations of general interest:—"If I am to sustain the Auditor's report on this matter one of two things seems to follow. Either (1) an *x*-ray specialist is to receive an additional remuneration for each *x*-ray plate which he takes, or (2) *x*-ray specialists are to be treated differently from heart specialists or eye specialists, or any other of the many kinds of specialists who are to be found in the medical profession. The first of these alternatives is certainly wrong, because it treats the specialist as both a photographer and a doctor, and remunerates him in the capacity of each. He is not a photographer. The special kind of photographic apparatus which he employs in his work is merely a highly specialised instrument for the ascertainment of facts to enable him to form an opinion on the case. It is nothing more than one of the modern scientific instruments in use in the profession. No better illustration of this can be found than in another production in this case. That was not a photograph, but a diagram on a sheet of paper. It was, as I understand, not made by the instrument itself, but by the specialist who worked it, and who sketched the diagram by manipulating the instrument—an orthoradiographic chart of the claimant's heart. This also was done by means of *x*-ray, but no photographer could have produced it. And it would surely be out of the question to treat that production as something to be paid for apart from the investigation made by the specialist, who, with knowledge which few of the profession have, was able to make and read the diagram and report on its meaning.

"The second alternative seems, at this time of day, to be equally unworthy of support. *X*-ray work, whatever it may have been a few years ago, is now by no means uncommon. And in cases under the Workmen's Compensation Act *x*-ray specialists are perhaps the most frequently employed of all. That is probably because they are able to ascertain the character of injuries to bones with precision and accuracy, which the ordinary practitioner cannot pretend to do. But a similar remark may be made generally of all specialists; emphatically it is so in the case of eye specialists, who also use instruments which only they can employ to their full power, and can by their aid discover what is hidden from the general practitioner. It seems to me that *x*-ray specialists cannot be put in a category by themselves to the effect of regarding them as entitled to higher remuneration than eye or ear or heart or any other specialists."

